# Metabolome, Plant Hormone, and Transcriptome Analyses Reveal the Mechanism of Spatial Accumulation Pattern of Anthocyanins in Peach Flesh

**DOI:** 10.3390/foods12122297

**Published:** 2023-06-07

**Authors:** Ping Sun, Chengkun Yang, Wencan Zhu, Jiaqi Wu, Xianrui Lin, Yi Wang, Jianxi Zhu, Chenfei Chen, Kaibing Zhou, Minjie Qian, Jiansheng Shen

**Affiliations:** 1 Jinhua Academy of Agricultural Sciences (Zhejiang Institute of Agricultural Machinery), Jinhua 321000, China; 2 Sanya Nanfan Research Institute of Hainan University, Sanya 572025, China; 3 Key Laboratory of Quality Regulation of Tropical Horticultural Crop in Hainan Province, Department of Horticulture, School of Horticulture, Haidian Campus, Hainan University, Haikou 570228, China

**Keywords:** *Prunus persica*, anthocyanins, metabolome, phytohormones, RNA-seq

## Abstract

Anthocyanins are important secondary metabolites in fruits, and anthocyanin accumulation in the flesh of peach exhibits a spatial pattern, but the relevant mechanism is still unknown. In this study, the yellow-fleshed peach, cv. ‘Jinxiu’, with anthocyanin accumulation in the mesocarp around the stone was used as the experimental material. Red flesh (RF) and yellow flesh (YF) were sampled separately for flavonoid metabolite (mainly anthocyanins), plant hormone, and transcriptome analyses. The results showed that the red coloration in the mesocarp was due to the accumulation of cyanidin-3-*O*-glucoside, with an up-regulation of anthocyanin biosynthetic genes (*F3H*, *F3′H*, *DFR*, and *ANS*), transportation gene *GST*, and regulatory genes (*MYB10.1* and *bHLH3*). Eleven *ERFs*, nine *WRKYs*, and eight *NACs* were also defined as the candidate regulators of anthocyanin biosynthesis in peach via RNA-seq. Auxin, cytokinin, abscisic acid (ABA), salicylic acid (SA), and 1-aminocyclopropane-1-carboxylic acid (ACC, ethylene precursor) were enriched in the peach flesh, with auxin, cytokinin, ACC, and SA being highly accumulated in the RF, but ABA was mainly distributed in the YF. The activators and repressors in the auxin and cytokinin signaling transduction pathways were mostly up-regulated and down-regulated, respectively. Our results provide new insights into the regulation of spatial accumulation pattern of anthocyanins in peach flesh.

## 1. Introduction

Anthocyanins are water-soluble pigments that not only contribute to the purple, blue, and red coloration of flowers and fruits but also protect plants against various biotic and abiotic stresses [1,2]. Due to their anti-oxidation capacity via scavenging reactive oxygen species (ROS), anthocyanins are considered as bioactive components for human health and, therefore, represent one of the most crucial aspects of fruit quality [3].

The biosynthesis of anthocyanins in plants is through the phenylpropanoid and flavonoid pathway [4]. Structural genes involved in anthocyanin biosynthesis includes *PAL* (phenylalanine ammonia lyase), *CHS* (chalcone synthase), *CHI* (chalcone isomerase), *F3H* (flavanone 3-hydroxylase), *F3′H* (flavonoid 3′-hydroxylase), *DFR* (dihydroflavonol 4-reductase), *ANS* (anthocyanidin synthase), and *UFGT* (UDP-glucose: flavonoid 3-*O*-glucosyltransferase). The expression of structural genes is regulated by MYB, bHLH, and WD40 transcription factors (TFs), which form a protein complex [5]. Anthocyanins are transported to the vacuole for storage after being synthesized in the cytoplasmic face of the endoplasmic reticulum, and the transporters include glutathione S-transferase (GST) and ATP-binding cassette (ABC) [6,7].

The accumulation of anthocyanins shows a spatial pattern. The spatial distribution in *Medicago truncatula* is regulated by a transcriptional repressor, MYB2 [8]. *myb2* mutants showed increased anthocyanin concentration in their hypocotyls and flowers, while the overexpression of *MYB2* inhibited anthocyanin accumulation in the roots. Anthocyanin accumulation in the taproots of radish shows differential spatial patterns among cultivars, resulting in cultivars with red skin and white flesh, green skin and pink-purple flesh, white skin and white flesh, and red skin and red flesh, which are correlated with the expression of anthocyanin biosynthetic genes, especially *RsUFGT* [9]. The spatial pattern variation of floral pigments between different *Mimulus* species is due to the competition between flavonols and anthocyanin biosynthesis, which is regulated by a R2R3-MYB TF [10].

Anthocyanin biosynthesis is controlled by plant hormones. Ethylene and abscisic acid (ABA) are involved in the ripening-related anthocyanin accumulation in climacteric fruits and non-climacteric fruits, respectively. Therefore, exogenous ethylene and ABA treatments usually promote anthocyanin accumulation in apple [11,12], grape [13,14], litchi [15], and mango [16,17]. However, ethylene has been reported to inhibit anthocyanin accumulation in pear [18,19] and peach [20]. Jasmonic acid (JA) is also widely used to promote anthocyanin biosynthesis in pear [21], apple [22], grape [23], and mango [17]. Cytokinin could also increase anthocyanin accumulation [24], while gibberellin (GA) represses anthocyanin biosynthesis [25]. Auxin could either promote or inhibit anthocyanin accumulation in different plant species [26,27,28,29].

A variety of peach, *Prunus persica* (L.) Batsch, is an economically important fruit crop in the temperate regions of the world. The flesh color of this peach exhibits white, yellow, or blood color, and blood flesh is due to the accumulation of anthocyanins. Anthocyanin biosynthesis in peach fruit is mainly regulated by PpMYB10.1, PpMYB10.2, and PpMYB10.3 [30,31], and in leaf, it is controlled by PpMYB10.4 [32]. The trait of blood fresh in the peach cv. ‘Dahongpao’ is controlled by a NAC TF, designated BLOOD (BL), which can form a heterodimer with PpNAC1 to activate the expression of *PpMYB10.1* and subsequent anthocyanin biosynthesis [33]. Apart from the blood-fleshed cultivars, some white- and yellow-fleshed cultivars also accumulate anthocyanins in the mesocarp around the stone, which is due to the high expression of *MYB10.1* and *MYB10.3* regulated by HY5 [31,34]. However, the molecular mechanism of anthocyanin accumulation in the inner part of the mesocarp, i.e., the regulation of the spatial expression pattern of *HY5*, is still unknown. Since the mesocarp around the stone is more affected by the plant hormones generated by the seed, our hypothesis is that plant hormones play a key role in the spatial accumulation pattern of anthocyanins in the flesh of peach.

**Figure 1:**
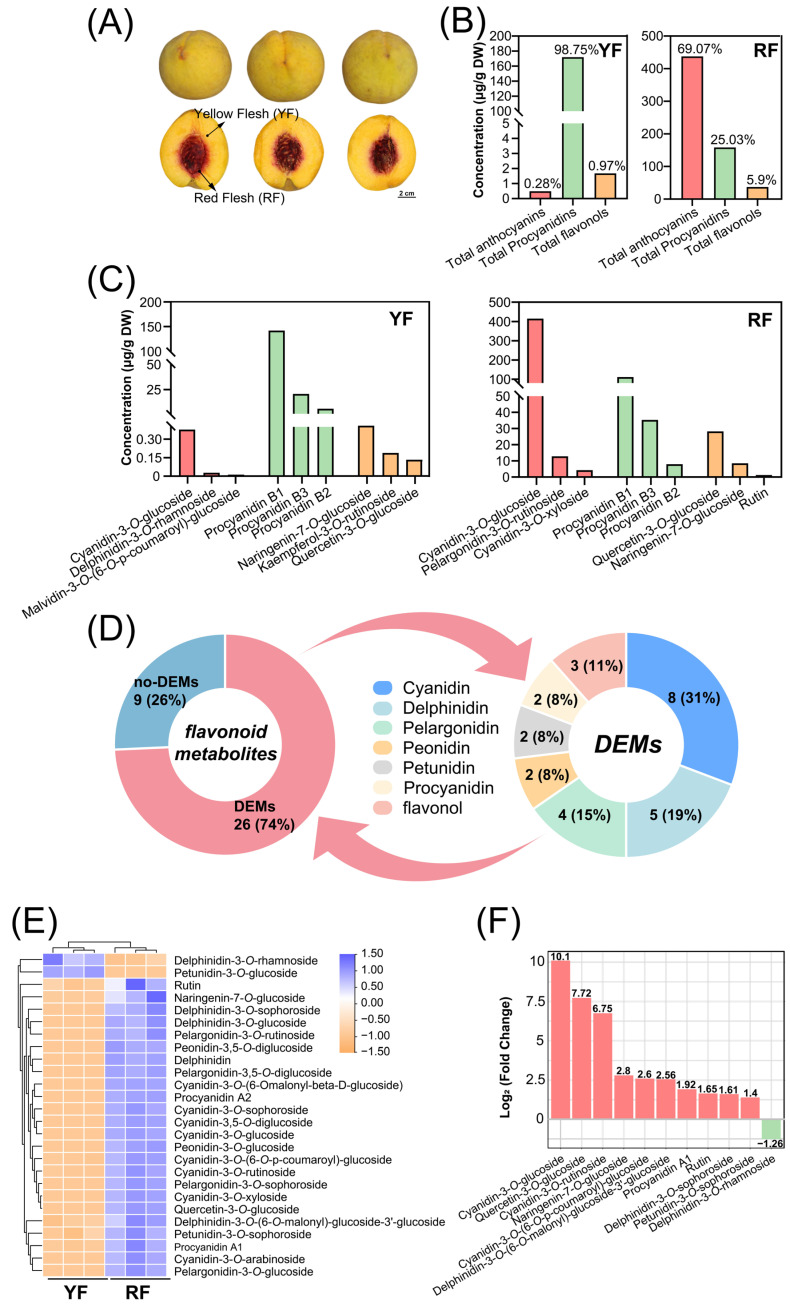
Phenotype and metabolomic profile in the flesh of ‘Jinxiu’ peach: (**A**) pigmentation pattern in the peel and flesh of ‘Jinxiu’ peach; (**B**) total anthocyanin, proanthocyanidin, and flavonol concentrations in the yellow flesh (YF) or red flesh (RF) of peach; (**C**) concentrations of predominant anthocyanin, proanthocyanidin, and flavonol compositions in the YF or RF of peach; (**D**) statistics of the expressed metabolites and differentially expressed metabolites; (**E**) heatmap of the 26 DEMs, and the color indicates the relative content of each DEM, ranging from yellow (low) to blue (high); and (**F**) summary of the DEMs with the most significant concentration fold change in the YF vs. RF.

In this study, the yellow-fleshed peach cv. ‘Jinxiu’ with anthocyanin accumulation around the stone was used as the experimental material (Figure 1A). The red inner part of the mesocarp around the stone and the yellow outer part of the mesocarp were sampled separately and subjected to anthocyanin metabolomic profiling, plant hormone measurement, and RNA-seq. In addition, the expressions of anthocyanin-related genes and plant hormone signal transduction genes were analyzed to screen for the key genes involved in the spatial distribution of anthocyanins in the flesh of the peach. This study will enrich our knowledge regarding the molecular mechanism of spatial accumulation of anthocyanins in fruits.

## 2. Results

### 2.1. Flavonoid Accumulation in Yellow Flesh and Red Flesh

In the yellow flesh, proanthocyanidins were the predominant flavonoids components, which accounted for 98.75% (171.9 µg/g of DW), while very little amount of flavonols and anthocyanins was detected (Figure 1B). The red flesh mainly accumulated anthocyanins, with a percentage of 69.07% (437.61 µg/g of DW), and certain amounts of proanthocyanidins (158.56 µg/g of DW) and flavonols (37.42 µg/g of DW) were detected, with a percentage of 25.03% and 5.9%, respectively (Figure 1B). The three main components of proanthocyanidins in the yellow flesh were procyanidins B1, B3, and B2, with a concentration of 142.53, 20.74, and 5.90 µg/g of DW, respectively, while no component of anthocyanins and flavonols reached a level higher than 2 µg/g of DW (Figure 1C). In the red flesh, cyanidin-3-*O*-glucoside, procyanidin B1, and quercetin-3-*O*-glucoside were the dominant components of anthocyanins, proanthocyanidins, and flavonols, with a concentration of 415.06, 111.69, and 28.2 µg/g of DW, respectively (Figure 1C).

In total, 35 components of flavonoids were detected, and 26 components were regarded as differentially expressed metabolites (DEMs) based on a threshold fold change ≥ 2 or a fold change ≤ 0.5 (Figure 1D). Among all the DEMs, 21 of them were anthocyanins, including eight cyanidins, five delphinidins, four pelargonidins, two peonidins, and two petunidins, and the rest included three flavonols and two procyanidins (Figure 1D). Overall, 24 out of the 26 DEMs were highly accumulated in the RF, with only delphinidin-3-*O*-rhamnoside and petunidin-3-*O*-glucoside mainly accumulated in the YF (Figure 1E). The three substances with the most tremendous difference in concentration in the RF and YF were cyanidin-3-*O*-glucoside, quercetin-3-*O*-glucoside, and cyanidin-3-*O*-rutinoside, with the value of Log_2_ (fold change) at 10.1, 7.72, and 6.75, respectively (Figure 1F).

### 2.2. RNA Sequencing

**Figure 2:**
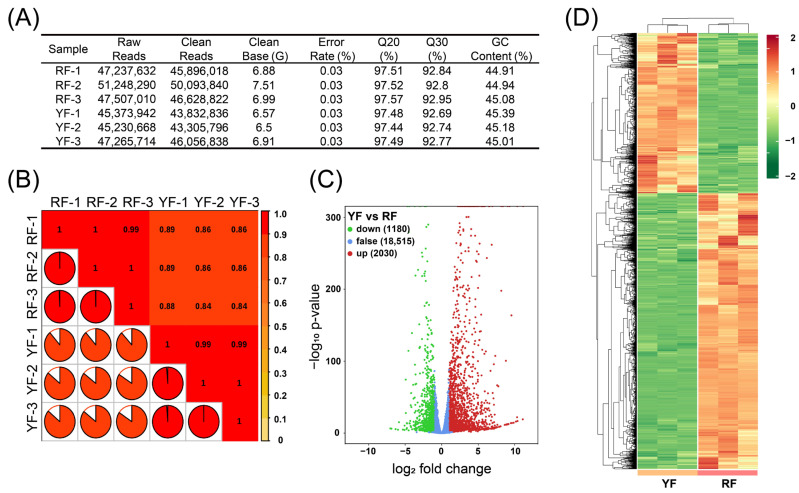
RNA-seq analysis results: (**A**) statistics on the quality and output of the RNA-seq libraries; (**B**) correlation of the samples, with the values indicating Pearson’s correlation coefficients; (**C**) volcano plots of the gene transcription profile in the YF and RF libraries, with the red dots representing up-regulated genes, the green dots representing down-regulated genes, and the blue dots representing non-differentially expressed genes; and (**D**) heatmap of the DEGs in YF and RF. Color indicates the expression level of each gene, from green (low) to red (high).

According to the RNA-seq, 45230668-51248290 raw reads were obtained in six samples, with 43305796-50093840 clean reads, and 6.5–7.51 G clean base (Figure 2A). The error rate was 0.03% among all the samples, and Q20, Q30, and GC contents were around 97.5%, 93%, and 45%, respectively (Figure 2A). The correlations among the three RF samples were almost 1, and the correlations among the three YF samples were 0.84–0.89 (Figure 2B). In total, 3210 differentially expressed genes (DEGs) were obtained, with 2030 highly expressed in the RF and 1180 highly expressed in the YF (Figure 2C,D).

**Figure 3:**
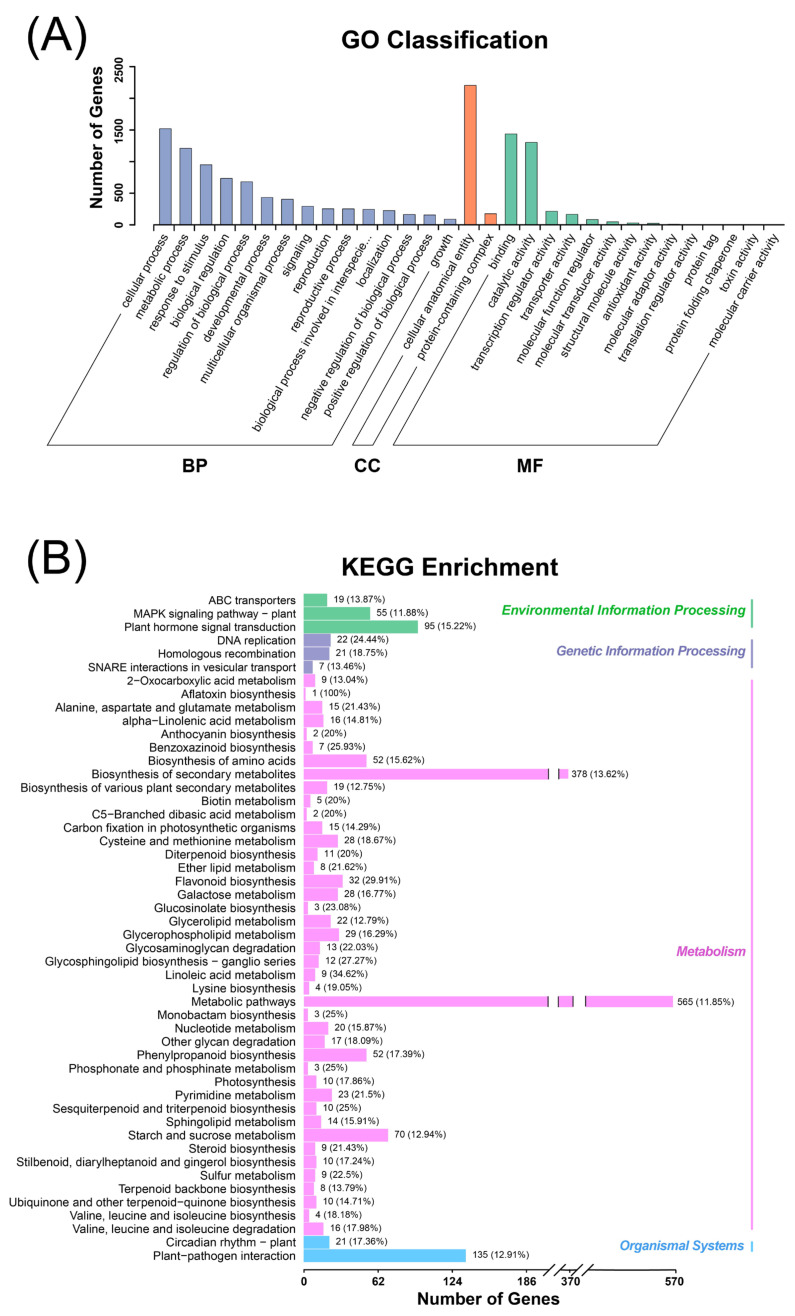
Analysis of DEGs: (**A**) gene ontology (GO) analysis of DEGs, and (**B**) Kyoto Encyclopedia of Genes and Genomes (KEGG) analysis of DEGs.

Based on the GO analysis, most differentially expressed genes were classified into biological process (BP) and molecular function (MF), while the highest number of genes were classified into cellular anatomical entity of cellular component (CC), with a total number of 2205 genes (Figure 3A). Most genes belonging to BP contributed to cellular process (1520 genes), followed by metabolic process (1210 genes), and response to stimulus (950 genes) (Figure 3A). Genes belonging to MF mainly participated in binding (1438 genes) and catalytic activity (1303 genes) (Figure 3A).

The KEGG analysis showed that most differentially expressed genes were classified into metabolism, including biosynthesis of secondary metabolites and metabolic pathways, with an enriched gene number of 565 and 378, respectively (Figure 3B). The other highly enriched pathways contain the MAPK signaling pathway-plant (55 genes), plant hormone signal transduction (95 genes), biosynthesis of amino acids (52 genes), phenylpropanoid biosynthesis (52 genes), starch and sucrose metabolism (70 genes), and plant–pathogen interaction (135 genes) (Figure 3B).

### 2.3. Expression of Anthocyanin Biosynthetic, Transportation, and Regulatory Genes

**Figure 4:**
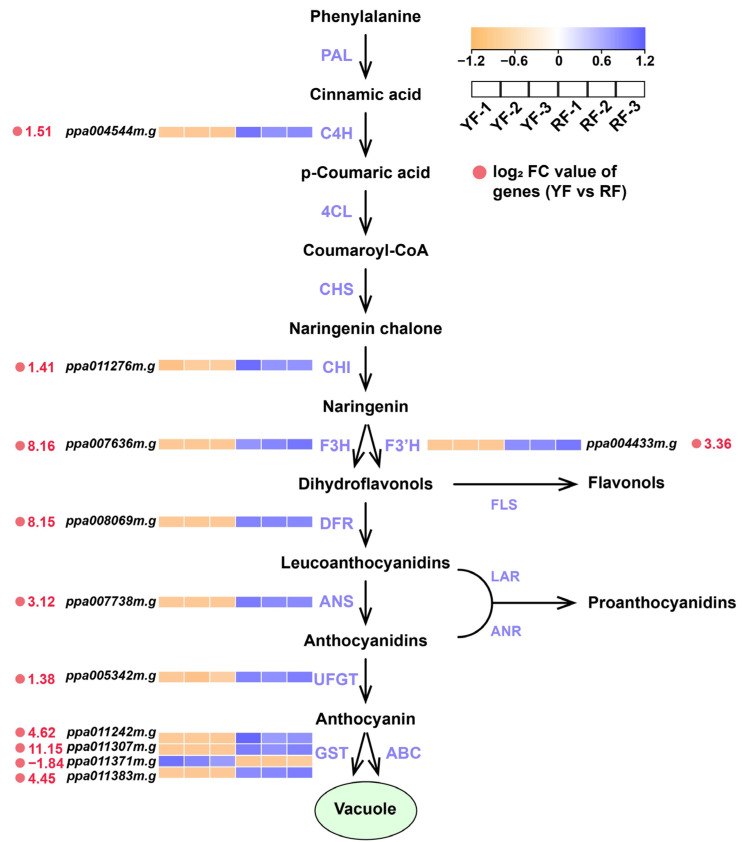
Expression pattern of the key candidate DEGs involved in anthocyanin biosynthesis and transportation in peach. The color scale ranging from yellow to blue represents the FPKM values from low to high. The values beside the gene IDs represent the log_2_ fold change values of the genes (YF vs. RF).

In order to select the key genes involved in anthocyanin biosynthesis, more strict thresholds were set for the DEGs, i.e., |log2FoldChange| > 1 and FPKM > 20. In total, 11 flavonoid biosynthetic and transportation DEGs were identified using RNA-seq, including one *C4H*, one *CHI*, one *F3H*, one *F3′H*, one *DFR*, one *ANS*, one *UFGT*, and four *GSTs* (Figure 4). Among these 11 genes, 10 genes were highly expressed in the RF, with only 1 *GST* transcript highly expressed in the YF (Figure 4). The genes highly expressed in the RF with a tremendous difference (fold change > 8, i.e., Log_2_ FC > 3) included *F3H* (Log_2_ FC = 8.16), *F3′H* (3.36), *DFR* (8.15), *ANS* (3.12), and three transcripts of *GST* (*ppa011242m.g*: 4.62; *ppa011307m.g*: 11.15; and *ppa011383m.g*: 4.45) (Figure 4).

**Figure 5:**
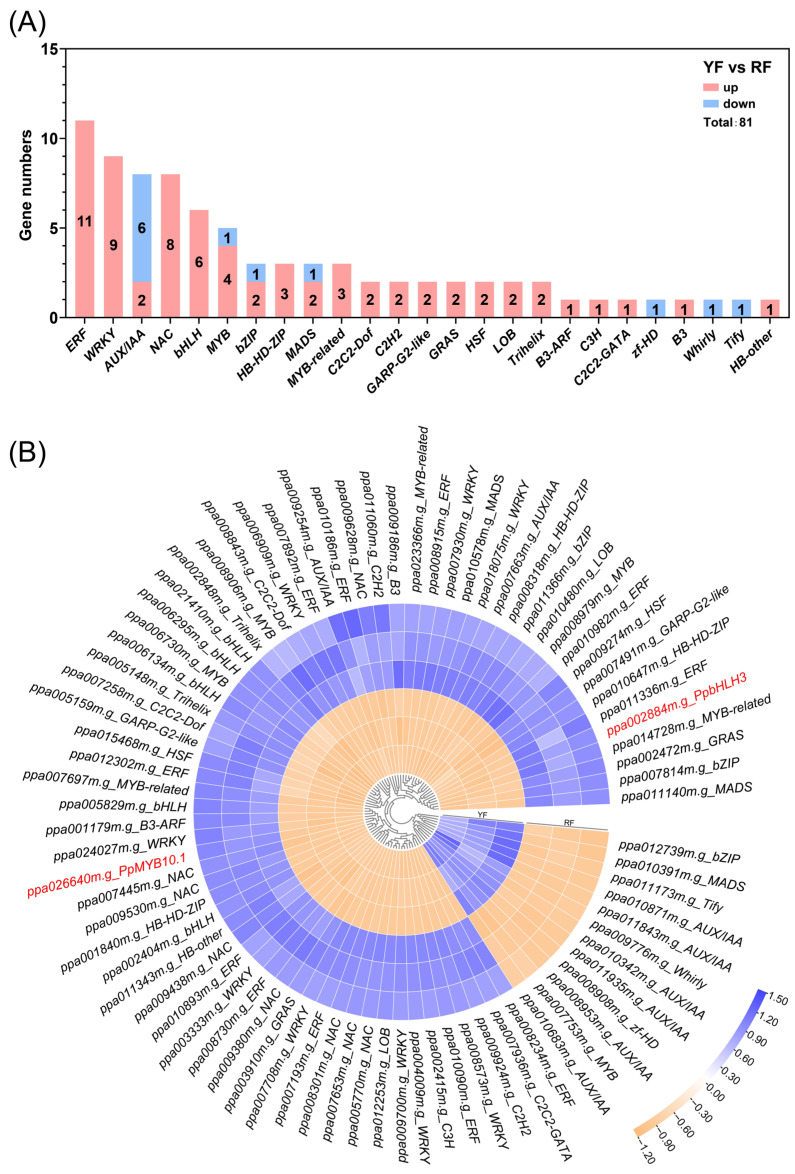
Regulatory genes involved in anthocyanin biosynthesis: (**A**) number of regulatory genes in different families, and (**B**) heatmap presenting the expression patterns of different regulatory genes in the YF and RF. The color scale ranging from yellow to blue represents the FPKM values from low to high. The genes highlighted by the red color are known key regulatory genes involved in anthocyanin biosynthesis, i.e., *PpMYB10.1* and *PpbHLH3*.

A total of 81 regulatory genes were identified via RNA-seq, and 69 (85.19%) of them were highly expressed in the RF (Figure 5A,B). The genes with the highest number of transcripts were *ERF* (11), followed by *WRKY* (9), *AUX/IAA* (8), *NAC* (8), *bHLH* (6), and *MYB* (5) (Figure 5A). Three transcripts of *bZIP*, *HB-HD-ZIP*, *MADS*, and *MYB-related* were identified, and two transcripts of *C2C2-Dof*, *C2H2*, *GARP-G2-like*, *GRAS*, *HSF*, *LOB,* and *Trihelix* were identified (Figure 5A). Most of the TFs highly expressed in the YF were from *AUX/IAA*, with a total number of six genes (Figure 5A). The previously reported *MYB* and *bHLH* involved in the regulation of anthocyanin biosynthesis in peach, i.e., *PpMYB10.1* and *PpbHLH3*, were also detected in this study, with a significantly higher expression level in the RF (Figure 5B).

### 2.4. Plant Hormone Distribution in Yellow Flesh and Red Flesh

**Figure 6:**
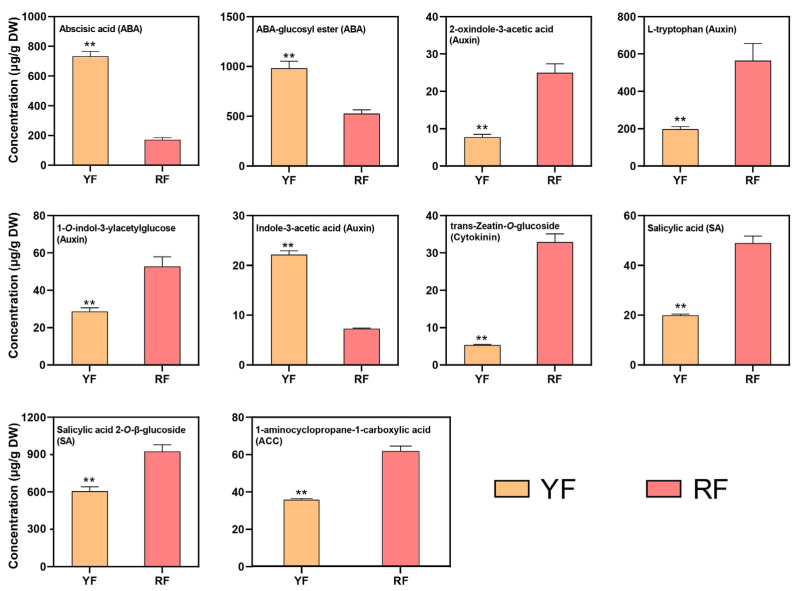
Plant hormone concentrations detected in the YF and RF of peach. The data are presented as the mean value ± standard deviation of three biological replicates. *p*-values of <0.01, are considered statistically significant and marked with two asterisks (**).

To ensure whether the spatial distribution pattern of anthocyanins in the flesh of peach was controlled by plant hormones, the red flesh and yellow flesh were subjected to plant hormone detection. In total, 10 components belonging to five plant hormones were enriched and deferentially distributed between the yellow flesh and red flesh (Figure 6). Most of the 10 DEMs belonged to auxin (4), followed by ABA (2) and SA (2), and the other 2 components belonged to cytokinin (1) and ACC (1) (Figure 6). ABA and indole-3-acetic acid were highly accumulated in the YF, while the rest of the plant hormones were highly distributed in the RF (Figure 6).

### 2.5. Expression of Plant Hormone Signal Transduction Genes

**Figure 7:**
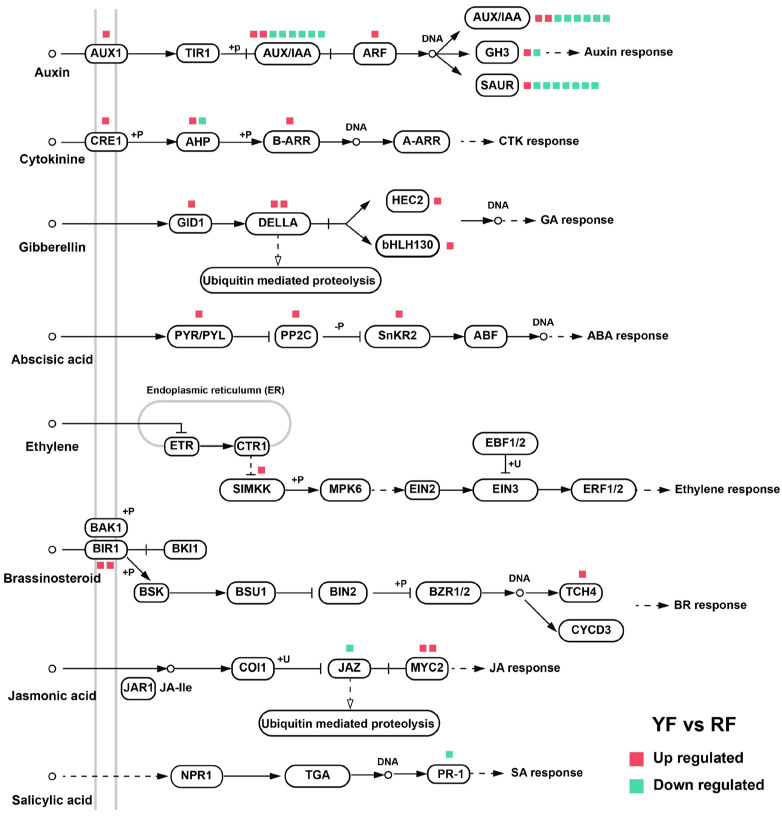
Categorization of the genes in different plant hormone signaling pathways. The genes are defined as being up-regulated (red box) or down-regulated (green box) based on the results for YF vs. RF.

For auxin signal transduction, most transcripts of *AUX/IAA* and *SAUR* were highly expressed in the YF, while higher expression levels of *AUX1* and *ARF* were detected in the RF (Figure 7). *PR-1*, which is involved in the salicylic acid signaling pathway, was also predominantly expressed in the YF (Figure 7). In contrast, genes participating in the signal transduction of other plant hormones were mainly highly expressed in the RF, including *CRE1* and *B-ARR* for cytokinin; *GID1*, *DELLA*, *HEC2*, and *bHLH130* for gibberellin; *PYR/PYL*, *PP2C*, and *SnKR2* for abscisic acid; *SIMKK* for ethylene; and *BIR1* and *TCH4* for brassinosteroid (Figure 7). For jasmonic acid signal transduction, *JAZ* was highly expressed in the YF, while *MYC2* was mainly transcribed in the RF (Figure 7).

### 2.6. Validation of DEGs Using qPCR

**Figure 8:**
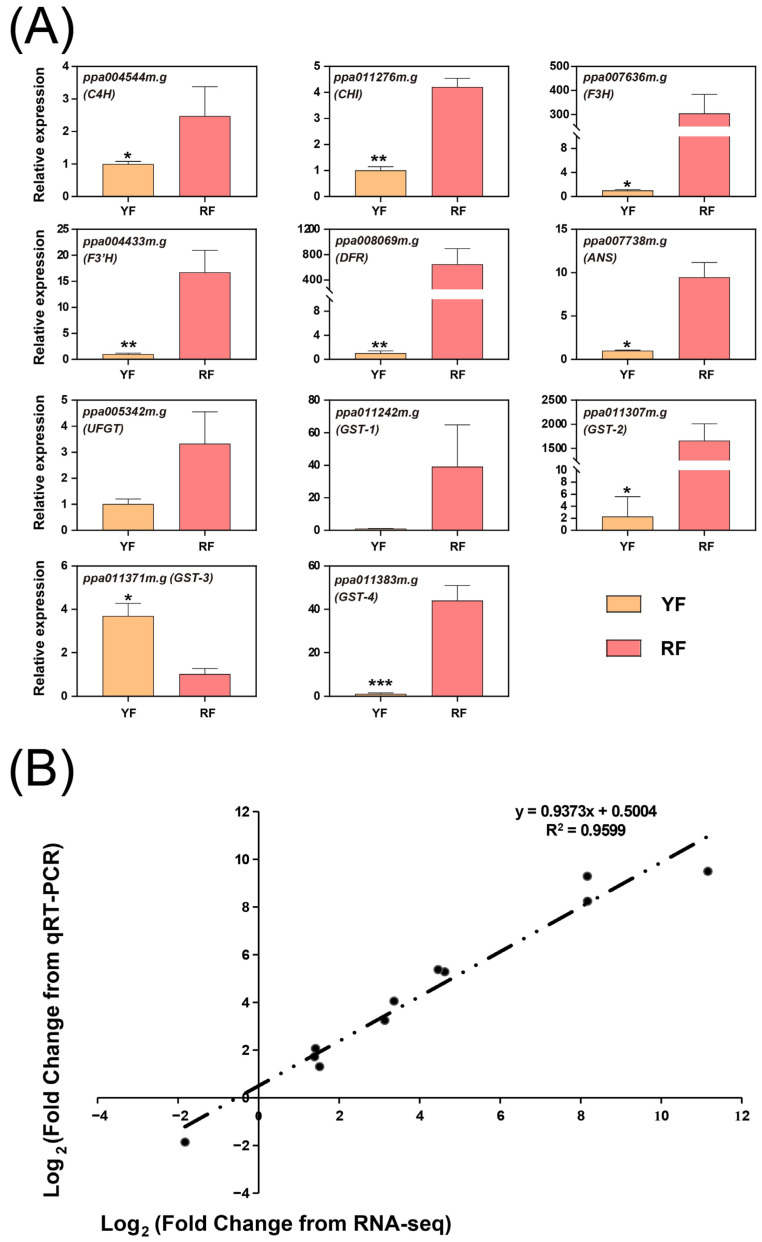
Validation of DEGs using qPCR. (**A**) Expression of genes related to anthocyanin biosynthesis and transportation based on qPCR. The data are presented as the mean value ± standard deviation of three biological replicates. *p*-values of <0.05, <0.01, and <0.001 are considered statistically significant and marked with one asterisk (*), two asterisks (**), and three asterisks (***), respectively. (**B**) Correlation analysis based on the RNA-seq data and qPCR.

To confirm the accuracy and reliability of the transcriptomic data, 11 anthocyanin-related genes were chosen and their expression was analyzed using qPCR (Figure 8A). The results of the transcriptome analysis and qPCR showed tremendous agreement, with a highly significant correlation coefficient of 0.9599 between the two approaches (Figure 8B).

## 3. Discussion

Peach fruit can accumulate anthocyanins in different parts, including its peel and mesocarp. Compared to other fruit species, peach is much more enriched with blood-fleshed germplasms and cultivars, such as ‘Dahongpao’ [33], ‘Tianjin Shui Mi’ [35], and ‘Wu Yue Xian’ [36]. However, yellow-fleshed or white-fleshed cultivars are more common, and quite many cultivars accumulate anthocyanins in the mesocarp around the stone, such as ‘Jinxiu’ in this study (Figure 1A), ‘Redhaven’, ‘Roza’, and ‘Fantasia’ [31]. Our results showed that the predominant anthocyanin component in ‘Jinxiu’ was cyanidin-3-*O*-glucoside (Figure 1C), which has also been reported in both red-peeled cultivars and red-fleshed cultivars [33,37]. All of these results indicate that cyanidin-3-*O*-glucoside is the dominant anthocyanin composition in peach, regardless of cultivars or tissues.

Among the structural genes, genes encoding enzymes in the middle of the anthocyanin biosynthetic pathway (*F3H* and *F3′H*), as well as late biosynthetic genes (LBGs) including *DFR* and *ANS*, were tremendously highly expressed in the RF (fold change > 8) (Figure 4). F3H is a key enzyme for flavonols and anthocyanin biosynthesis by catalyzing the conversion of naringenin to dihydroflavonols, and RNAi-mediated silencing of *F3H* can greatly reduce anthocyanin accumulation in strawberry fruit and carnation flower [38,39]. F3′H catalyzes hydroxylation at the 3′ position of dihydrokaempferol B-ring, which is essential for the formation of cyanidin. Cyanidin accumulation in the peel of the red apple cultivar ‘Red Delicious’ is highly correlated with the expression of *F3′H*, and the ectopic expression of apple *F3′H* leads to the high accumulation of cyanidin in Arabidopsis seedling and tobacco flower [40]. Anthocyanin biosynthesis is always associated with an up-regulation of LBGs, including *DFR*, *ANS*, and *UFGT*, in various plant species, including pear [41,42,43], apple [11,40], peach [31,33], and mango [44,45]. Surprisingly, compared to *DFR* and *ANS*, the up-regulation of *UFGT* in the RF was much more moderate (Figure 4), indicating that UFGT was not the limiting factor for anthocyanin accumulation in the YF. GST is crucial for the transportation of anthocyanins to vacuoles. In peach, a *GST* gene (also known as *Riant*) regulating anthocyanin biosynthesis in both flower and fruit has been cloned [46,47,48], which corresponds with *ppa011307m.g* in this study, with an expression 2272 times higher in the RF than in the YF (Figure 4). Apart from *ppa011307m.g*, the other two transcripts of *GST*, i.e., *ppa011242m.g* and *ppa011383m.g*, were also greatly up-regulated in the RF (Figure 4), indicating the synergetic function of GST family members during anthocyanin biosynthesis. All of these results suggest that the up-regulated expressions of biosynthetic and transportation genes are necessary for anthocyanin accumulation and mesocarp coloration in peach.

For the TFs identified by RNA-seq, 85.19% of them were highly expressed in the RF (Figure 5A,B), indicating positive regulation was predominant during anthocyanin biosynthesis. *MYB* and *bHLH*, which are known for regulating anthocyanin biosynthesis in peach, i.e., *PpMYB10.1* and *PpbHLH3*, were also significantly highly expressed in the RF (Figure 5B). Anthocyanin biosynthesis in peach flesh is mainly regulated by *PpMYB10.1*, *PpMYB10.2*, *PpMYB10.3*, *PpbHLH3*, and *PpbHLH33*, which show divergent functions. Anthocyanin accumulation in the mesocarp around the stone in ‘Redhaven’, ‘Roza’, and ‘Fantasia’ peach cultivars is regulated by *PpMYB10.1*, *PpMYB10.3*, and *PpbHLH3*, and the overexpression of *PpMYB10.1/PpbHLH3* and *PpMYB10.3/PpbHLH3* could promote anthocyanin accumulation in tobacco leaf [31]. The expression of *PpMYB10.1*, but not *PpbHLH3*, is always highly correlated with anthocyanin concentration in peach [33,47,49], while the co-expression of *bHLH* is necessary for *MYB*-induced anthocyanin accumulation [50]. Eleven *ERFs*, nine *WRKYs*, and eight *NACs* were also regarded as the candidate genes regulating anthocyanin accumulation in peach in this study (Figure 5), and among them, NAC has been shown to promote anthocyanin biosynthesis in peach by activating the expression of *MYB* [33,51]. ERF and WRKY have been widely reported to regulate anthocyanin biosynthesis by promoting the expression of anthocyanin-related genes [52,53] or through protein–protein interaction with MYB [53,54,55]. Our results indicate that anthocyanin accumulation in the mesocarp around the stone of ‘Jinxiu’ peach is probably regulated by PpMYB10.1 and PpbHLH3, and TFs, such as ERF, WRKY, and NAC, could also be potential contributors.

The predominant components of auxin, cytokinin, ACC, and salicylic acid were highly accumulated in the RF (Figure 6), with an up-regulation of the key activators in the auxin and cytokinin signaling pathway, and a down-regulation of most transcripts of the auxin signaling repressor *AUX/IAA* (Figure 7), indicating the positive role of auxin and cytokinin in peach anthocyanin accumulation. The role of auxin in regulating anthocyanin biosynthesis in different fruit species is divergent. Exogenous auxin treatment inhibits anthocyanin accumulation in apple and raspberry [26,27] but promotes anthocyanin biosynthesis in sweet cherry and peach [28,29]. Cytokinin induces anthocyanin accumulation in pear and apple through type-B response regulators (B-RRs) and the repressor MdMYBL2, respectively [24,56]. Surprisingly, a higher ABA concentration was detected in the YF, and both activators (*PYR/PYL* and *SnKR2*) and repressor (*PP2C*) in the ABA signaling pathway were up-regulated (Figure 6 and Figure 7), making the results a bit confusing. ABA is widely used to induce anthocyanin biosynthesis in fruits, including apple, litchi, and sweet cherry, through the up-regulation of *MdbZIP44* [12], *LcMYB1* [57], and *PacMYBA* [58]. Taking all the results together, auxin and cytokinin are the candidate plant hormones regulating anthocyanin biosynthesis in peach.

## 4. Materials and Methods

### 4.1. Plant Materials

The fruits of ‘Jinxiu’ peach were obtained from a commercial orchard in Jindong District, Jinhua City, Zhejiang Province, China. Three mature trees with similar size and uniform exposure to sunlight were selected, and one tree was regarded as one biological replicate. For each tree, 4 mature fruits were harvested and mixed together, and the yellow flesh in the outer part of the mesocarp and the red flesh in the inner part of the mesocarp were sampled separately with liquid nitrogen and stored at −80 °C.

### 4.2. Metabolomic Profiling

The metabolomic profiling of flavonoids (mainly anthocyanins) was performed by Metware Biotechnology Co., Ltd. (Wuhan, China), and the details have been described in our previous study [44]. Briefly, 0.05 g of freeze-dried flesh sample was extracted twice with 500 µL of 50% methanol containing 0.1% HCl, and the supernatants obtained at two different times were mixed together, filtered through a microporous membrane (0.22 µm), and stored in a vial for subsequent high-performance liquid chromatography with tandem mass spectrometric (HPLC−MS/MS) analysis. The mass spectrometry-detected flavonoid compounds were identified according to the Metware Database (MWDB). The substance concentration was calculated using the linear equation of corresponding standard based on the chromatographic peak area.

### 4.3. Plant Hormone Measurement

The quantification of endogenous plant hormones, including auxin, cytokinin, abscisic acid, jasmonate, salicylic acid, gibberellin, strigolactone, and 1-aminocyclopropane-1-carboxylic acid (ACC, ethylene precursor), was performed by Metware Biotechnology Co. Ltd. (Wuhan, China) using an LC–MS/MS platform, which has been described by Guo et al. [59].

### 4.4. RNA Extraction and Sequencing

The methods for RNA extraction and sequencing have been described in our previous paper [45]. The clean reads were mapped to the reference genome of the peach cv. ‘Lovell’ (https://www.rosaceae.org/species/prunus_persica/genome_v1.0, accessed on 1 March 2023) using TopHat [60]. Cufflinks was used to assemble the transcripts from the reads, and gene expression was calculated and exhibited as fragments per kilobase of transcript per million fragments mapped (FPKM). Differential expression between two groups was analyzed using the DESeq R package (1.10.1), and genes with |log_2_ fold change|> 1 and a significant *p*-value < 0.05 were regarded as differentially expressed genes (DEGs). The raw data of the RNA-seq were submitted to NCBI with the following ID number: PRJNA945801.

### 4.5. cDNA Synthesis and Quantitative Real-Time PCR

The protocols for cDNA synthesis and quantitative real-time PCR (Q-PCR) have been described by Qian et al. [45]. The Q-PCR primers for analyzing the expression of anthocyanin biosynthetic and transportation genes were designed using primer 3.0 (https://bioinfo.ut.ee/primer3-0.4.0/, accessed on 20 March 2023), and listed in Supplementary File S1. The peach *PpN1* gene (GDR accession no. ppa009483m) was selected as a constitutive control according to a previous report [31].

### 4.6. Statistical Analysis

The experimental data were subjected to Student’s *t*-test using SPSS 27.0 (SPSS, Chicago, IL, USA) to analyze for statistical differences between the red flesh and yellow flesh. Probability values of <0.05, <0.01, and <0.001 were considered statistically significant and marked with one asterisk (*), two asterisks (**), and three asterisks (***), respectively.

## 5. Conclusions

Cyanidin-3-*O*-glucoside is the predominant anthocyanin component in ‘Jinxiu’ peach, which is highly accumulated in the red mesocarp around the stone while almost undetectable in the yellow mesocarp. The RNA-seq showed that the expression of anthocyanin biosynthetic genes (*F3H*, *F3′H*, *DFR,* and *ANS*), transportation gene *GST*, and regulatory genes (*MYB10.1* and *bHLH3*) were significantly correlated with anthocyanin accumulation. Other TFs, including ERF, WRKY, and NAC, were also candidate regulators of anthocyanin biosynthesis in peach. Higher concentrations of the predominant auxin and cytokinin components were detected in the RF, with the up-regulation of several activators and down-regulation of a repressor in the signaling transduction pathway. Our results provide a comprehensive analysis and a broad overview of the spatial accumulation pattern of anthocyanins in peach.

## Data Availability

The reported data can be found in the Supplementary Materials. The raw data of the RNA-seq have been submitted to NCBI with the following ID number: PRJNA945801.

## Supplementary Materials

The following supporting information can be downloaded at https://www.mdpi.com/article/10.3390/foods12122297/s1. File S1: Sequences of the primers used in this study.
